# WS-SfMLearner: self-supervised monocular depth and ego-motion estimation on surgical videos with unknown camera parameters

**DOI:** 10.1117/1.JMI.12.2.025003

**Published:** 2025-04-30

**Authors:** Ange Lou, Jack Noble

**Affiliations:** Vanderbilt University, Department of Electrical and Computer Engineering, Nashville, Tennessee, United States

**Keywords:** self-supervised learning, unknown camera, depth estimation, ego-motion estimation

## Abstract

**Purpose:**

Accurate depth estimation in surgical videos is a pivotal component of numerous image-guided surgery procedures. However, creating ground truth depth maps for surgical videos is often infeasible due to challenges such as inconsistent illumination and sensor noise. As a result, self-supervised depth and ego-motion estimation frameworks are gaining traction, eliminating the need for manually annotated depth maps. Despite the progress, current self-supervised methods still rely on known camera intrinsic parameters, which are frequently unavailable or unrecorded in surgical environments. We address this gap by introducing a self-supervised system capable of jointly predicting depth maps, camera poses, and intrinsic parameters, providing a comprehensive solution for depth estimation under such constraints.

**Approach:**

We developed a self-supervised depth and ego-motion estimation framework, incorporating a cost volume–based auxiliary supervision module. This module provides additional supervision for predicting camera intrinsic parameters, allowing for robust estimation even without predefined intrinsics. The system was rigorously evaluated on a public dataset to assess its effectiveness in simultaneously predicting depth, camera pose, and intrinsic parameters.

**Results:**

The experimental results demonstrated that the proposed method significantly improved the accuracy of ego-motion and depth prediction, even when compared with methods incorporating known camera intrinsics. In addition, by integrating our cost volume–based supervision, the accuracy of camera parameter estimation, including intrinsic parameters, was further enhanced.

**Conclusions:**

We present a self-supervised system for depth, ego-motion, and intrinsic parameter estimation, effectively overcoming the limitations imposed by unknown or missing camera intrinsics. The experimental results confirm that the proposed method outperforms the baseline techniques, offering a robust solution for depth estimation in complex surgical video scenarios, with broader implications for improving image-guided surgery systems.

## 1 Introduction

Depth estimation from surgical images plays an important role in the field of image-guided surgery, such as three-dimensional (3D) reconstruction,^1^ navigation, and augmented reality. Depth estimation from surgical video frames is a challenging task due to several factors such as non-Lambertian reflection properties of tissues, motion blur, and lack of photometric consistency across frames.^2^ However, recent advances in computer vision and deep learning techniques have shown promising results in addressing these challenges. Two popular approaches for depth estimation from a sequence of single-camera surgical images are structure-from-motion (SfM)^3^ and shape-from-shading,^4^ which are two approaches that use classical stereo reconstruction methods between frames acquired at two time points from two different camera positions relative to the scene. Another approach is to use monocular depth estimation, which relies on learning-based methods to infer a depth map of the scene from a single image.

**Fig. 1:**
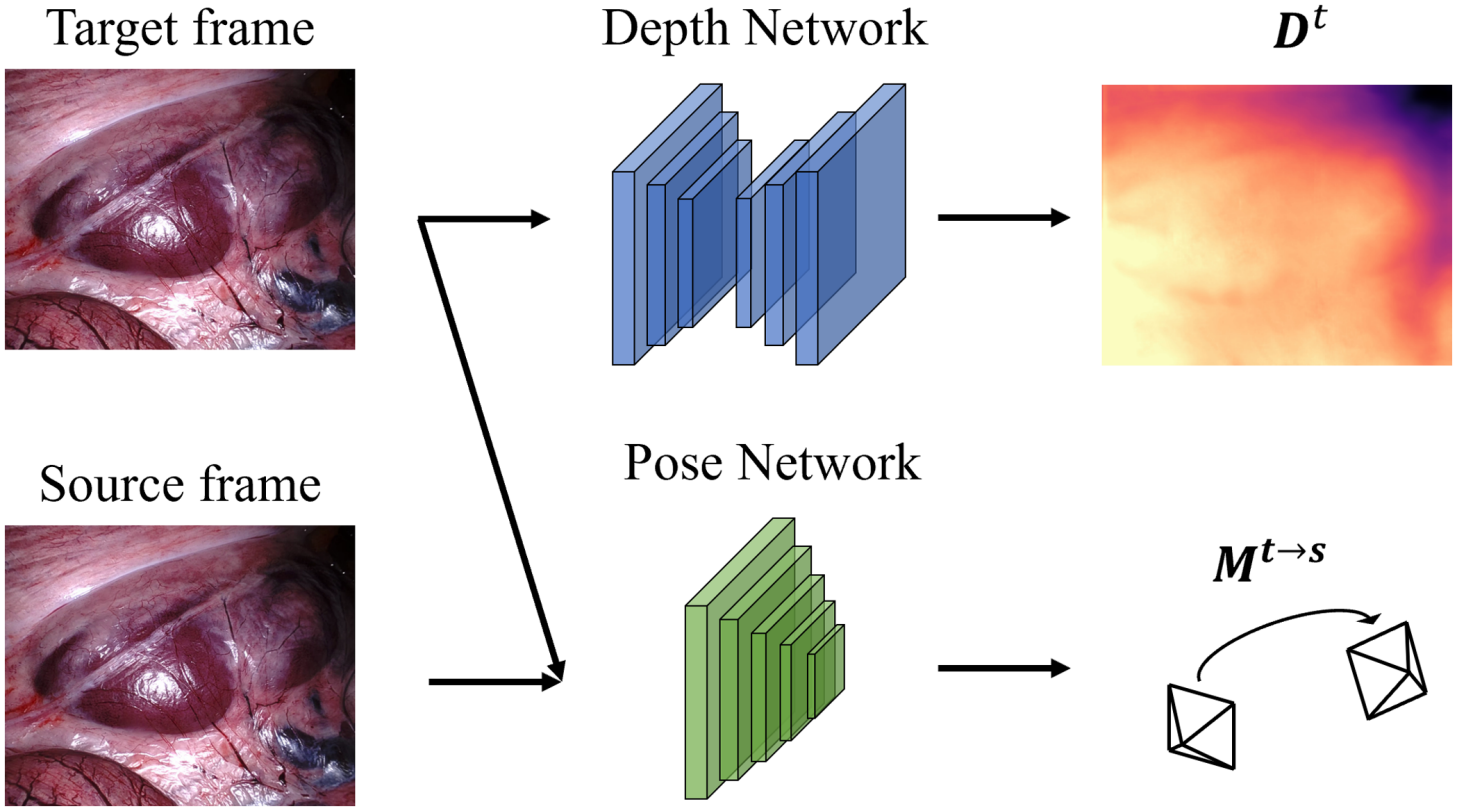
SfM-inspired system consists of a depth network and pose network used to predict the depth map for the target frame and motion (pose) from target to source, respectively.

Recently, deep neural networks have shown effectiveness in monocular video frame depth estimation. Fully supervised approaches, e.g., Xu et al.,^5^ Cao et al.,^6^ and Fu et al.,^7^ have achieved outstanding results. Unfortunately, it is difficult to collect large-scale and accurate surgical video depth map datasets due to the difficulty in creating a ground truth. Thus, few such public datasets are available. As a result, many promising self-supervised monocular depth and camera ego-motion estimation networks are proposed to solve this dilemma, such as SfMLearner,^8^ Monodepth2,^9^ SC-SfMLearner,^10^ and Endo-SfM.^11^ These self-supervised methods use disparity information among adjacent frames to supervise the neural networks and can produce relative depth maps. Relative depth maps do not encode exact depth but rather the relative distance to the camera among different objects in the scene and have been shown to improve 3D scene understanding.^12^ The common element of these SfM-inspired neural network systems is to predict the depth and ego-motion simultaneously and then to warp source frames to target frames using the coordinate transformation represented in Eq. (1)

$$h(p^{s\to t})=[K|0]M^{t\to s}[\begin{array}{c} D^{t}K^{-1}h(p^{t})\\ 1 \end{array}],$$ (1)

where $h(p^{t})$ and $h(p^{s\to t})$ are the homogeneous pixel coordinates in target frame t and those from target frame t mapped to source frame s, and K, $D^{t}$, and $M^{t\to s}$ are transformations representing the camera intrinsic parameters, the depth map of target frame, and the camera ego-motion from t to s, respectively. The architecture of this type of neural network system is shown in Fig. 1.

Various types of SfM learner systems have successfully solved the major challenges of surgical video analysis, such as motion blur, specular reflection, and regions with homogeneous content. However, to achieve promising performance by unsupervised methods, a large dataset is crucial. There are many surgical video datasets^13^^,^^14^ that do not provide the camera intrinsic parameters or camera motion data, which prevent using those data to train existing unsupervised depth estimation networks. Gordon et al.^15^ overcame this challenge when using unknown source data from natural scenes by first using a convolutional neural network (CNN) to predict the camera intrinsic parameters from unlabeled data. Although Gordon’s method achieves promising results on depth map and camera intrinsic parameters estimation, the accuracy of predicted camera intrinsic parameters was shown to be highly dependent on the dataset itself. For example, a dataset with more camera rotations enables more accurate camera intrinsic prediction.^15^ Therefore, we hypothesize that a camera intrinsic self-supervision strategy can improve the depth and ego-motion estimation performance. However, it is more challenging to predict accurate depth maps and camera intrinsic parameters in the unknown surgical scene than in natural images due to the limitation of labeled data and the challenges involved with surgical video analysis discussed above.

Our contributions are as follows: To the best of our knowledge, our proposed method, which we call “WS-SfMLearner” for wild, surgical SfMLearner, is the first that estimates the relative depth of the surgical scene and camera ego-motion using monocular camera video frames with unknown camera positions and intrinsic parameters. Further, we design a cost volume–based camera intrinsic parameter self-supervision method to train a CNN-based camera intrinsic parameter prediction module. This leads to improved relative depth and ego-motion estimate with WS-SfMLearner. Because WS-SfMLearner does not rely on known camera positions, intrinsic parameters, or stereo images, it is widely applicable to many surgical video datasets.

## 2 Related Work

In this section, we review relevant self-supervised deep learning–based methods in depth estimation. SfMLearner^8^ developed the self-supervised framework to solve the unsupervised depth prediction problem as a warping–based view synthesis task. After that, many works improve the performance of SfMLearner by exploring geometric constraints,^10^^,^^16^ designing representation learning components,^17^^,^^18^ and applying attention mechanisms.^19^ Although these methods work well in autonomous driving areas, they are not generally applicable to surgical environments.

Recently, Turan et al.^20^ first proposed a self-supervised depth and ego-motion estimation system in endoscope scenes. After that, many works exploring methods to enhance the photometric robustness such as Endo-SfMLearner^11^ used an affine brightness transformer and AF-SfMLearner^21^ introduced appearance flow. However, seldom studies solve the longstanding challenge—unknown source video depth and ego-motion estimation. Gordon et al.^15^ first solved this problem by introducing a camera intrinsic CNN, which predicts camera parameters during training. However, later works^22^^,^^23^ do not investigate self-supervision methods to improve camera intrinsic estimation.

## 3 Method

In this section, we first introduce the baseline self-supervised system we build upon—AF-SfMLearner,^8^ a structure-from-motion–inspired neural network with appearance flow designed to mitigate illumination interference in surgical scenes (https://github.com/ShuweiShao/AF-SfMLearner). Then, we show the details of the camera intrinsic prediction CNN we propose. Finally, we introduce the cost volume–based method to supervise the camera CNN training.

### 3.1 Baseline: AF-SfMLearner

**Fig. 2:**
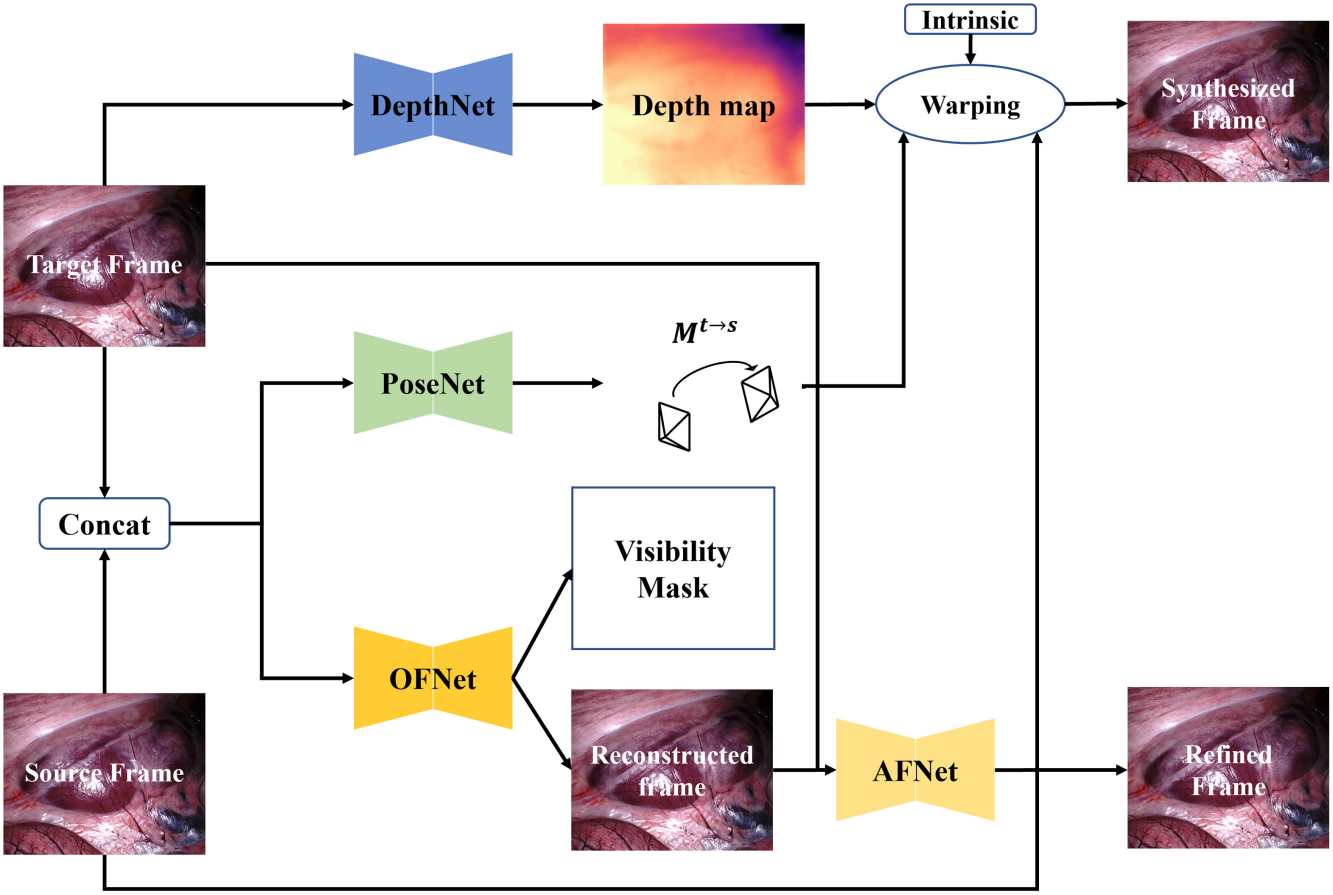
Architecture of the AF-SfMLearner.

We adopt AF-SfMLearner as the baseline, as shown in Fig. 2, containing a depth network (DepthNet) and pose network (PoseNet) to predict the depth map of the target frame $\left(I^{t}(p)\right)$ and ego-motion from the target to the source frame $\left(I^{s}(p)\right)$. In addition, AF-SfMLearner also uses OFNet to predict the optical flow between the target and the source frame. The optical flow is used to generate the source-to-target frame and the visibility mask. Then, the original target frame and source-to-target are sent into AFNet to do brightness calibration to generate a refined target frame.

The system is supervised by two losses as shown in Eq. (2)

L=D(p)+κR(p), (2)

where D(p) and R(p) are data fidelity and a Tikhonov regularizer, respectively. The κ is the loss weight to balance these two items

$$D(p)=\underset{p}{\sum }V(p)*\Phi (I^{s\to t}(p),I^{t}(p)+C(p)),$$ (3)

where V(p) is the visibility mask generated by OFNet, and $I^{s\to t}(p)$ is the synthesized target frame. Here, we choose two nearby frames of $I^{t}(p)$ as $I^{s}(p)$, $I^{s}(p)\in \{I^{t-1}(p),I^{t+1}(p)\}$. C(p) is the brightness calibration image used to generate refined target frames. Finally, $\Phi (I^{s\to t},I^{t})=\alpha \frac{1-\mathrm{SSIM}(I^{s\to t},I^{t})}{2}+(1-\alpha )|I^{s\to t}-I^{t}{|}_{1}$.

The Tikhonov regularizer R(p) contains three parts as shown in Eq. (4)

$$R(p)=\lambda_{1}L_{\mathrm{rs}}+\lambda_{2}L_{\mathrm{ax}}+\lambda_{3}L_{\mathrm{es}},$$ (4)

where $L_{\mathrm{rs}}$, $L_{\mathrm{ax}}$, and $L_{\mathrm{es}}$ are shown in Eqs. (5)–(7), respectively

$$L_{\mathrm{rs}}=\underset{p}{\sum }|\nabla C(p)|*e^{-\nabla |I^{t}(p)-I^{s\to t}(p)|}.$$ (5)

$L_{\mathrm{rs}}$ is defined as residual-based smoothness loss to encourage the AFNet to generate a smooth appearance flow map

$$L_{\mathrm{ax}}=\underset{p}{\sum }V(p)*\Phi (I_{c}^{s\to t}(p),I^{t}(p)+C(p)).$$ (6)

$L_{\mathrm{ax}}$ is designed to provide auxiliary supervision for AFNet. The difference between $L_{\mathrm{ax}}$ and D(p) is $I_{c}^{s\to t}(p)$ representing the reconstructed target frames by optical flow from OFNet in $L_{\mathrm{ax}}$

$$L_{\mathrm{es}}=\underset{p}{\sum }|\nabla D(p)|*e^{-\nabla |I^{t}(p)|}.$$ (7)

The edge-aware smoothness loss $L_{\mathrm{es}}$ is designed to encourage smoother depth map predictions.

### 3.2 Camera Intrinsic CNN

**Fig. 3:**
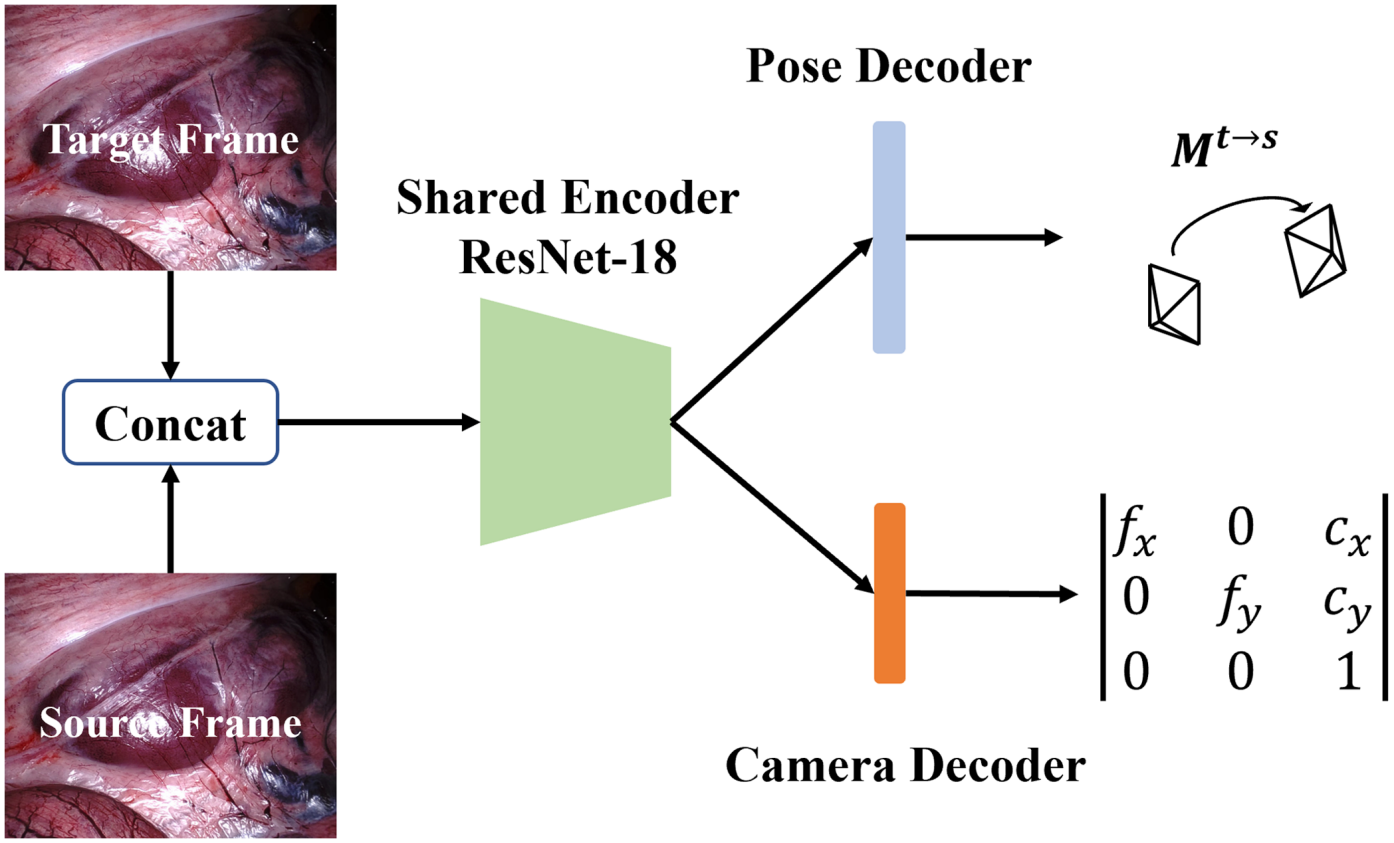
Architecture of the camera intrinsic prediction CNN.

The architecture of camera intrinsic CNN is shown in Fig. 3. We utilize the pre-trained ResNet-18^24^ as an encoder, followed by a pose decoder and a camera decoder to predict the ego-motion and intrinsic matrix, respectively. In the camera decoder route, the feature from the encoder passes through a convolution layer and average pooling layer then to the focal length convolution and offset convolution layer to generate the focal length and principal point offset of camera intrinsic parameters.

### 3.3 Cost Volume

**Fig. 4:**
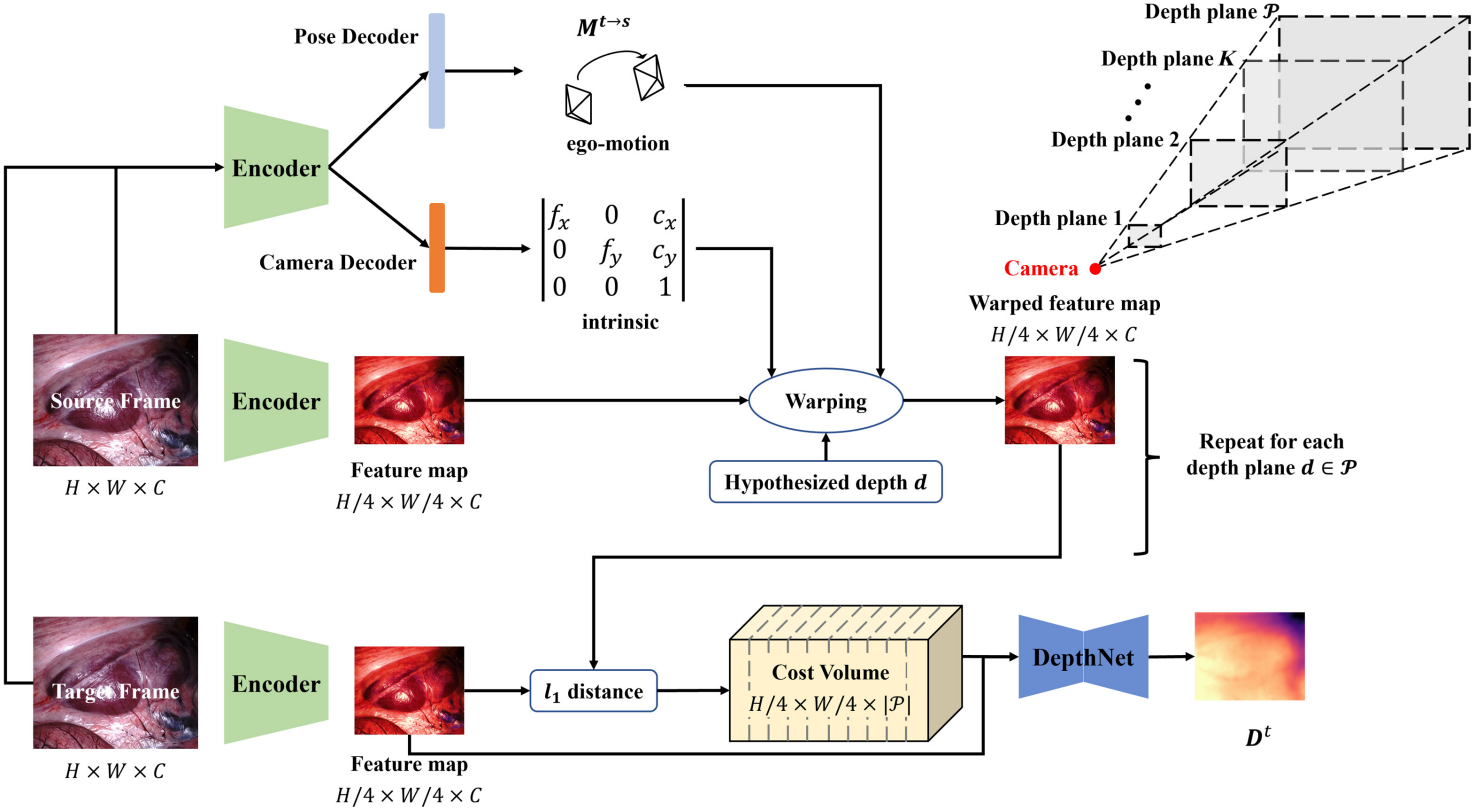
Architecture of the cost volume module.

Inspired by ManyDepth,^25^ we leverage the cost volume module to measure the geometric compatibility at different depth values between the pixels from target and source frames from the input videos. The cost volume module architecture is shown in Fig. 4. It shares the same PoseNet and CameraNet with AF-SfMlearner to generate ego-motion and camera intrinsic parameters. Then, we also use ResNet-18 as the encoder to extract the feature maps of target and source frames. In cost volume computing, we only use past frames as nearby views of target frames, $I^{s}(p)\in \{I^{t-1}(p),\ldots ,I^{t-N}(p)\}$. Then, we define a set of ordered depth planes P, each perpendicular to the optical axis at $I^{t}$. The depth planes are linearly distributed between $d_{\min}$ and $d_{\max}$. After warping source feature maps with predicted ego-motion, intrinsic parameters, and defined depth plane to the target domain, we calculate the $l_{1}$ distance between the target feature map $f^{t}$ and synthesized the target feature map $f^{s\to t}$ to build the cost volume. Then, we use DepthNet to predict the target depth map from the cost volume, which permits the network to leverage inputs from multiple viewpoints.

### 3.4 WS-SfMLearner

**Fig. 5:**
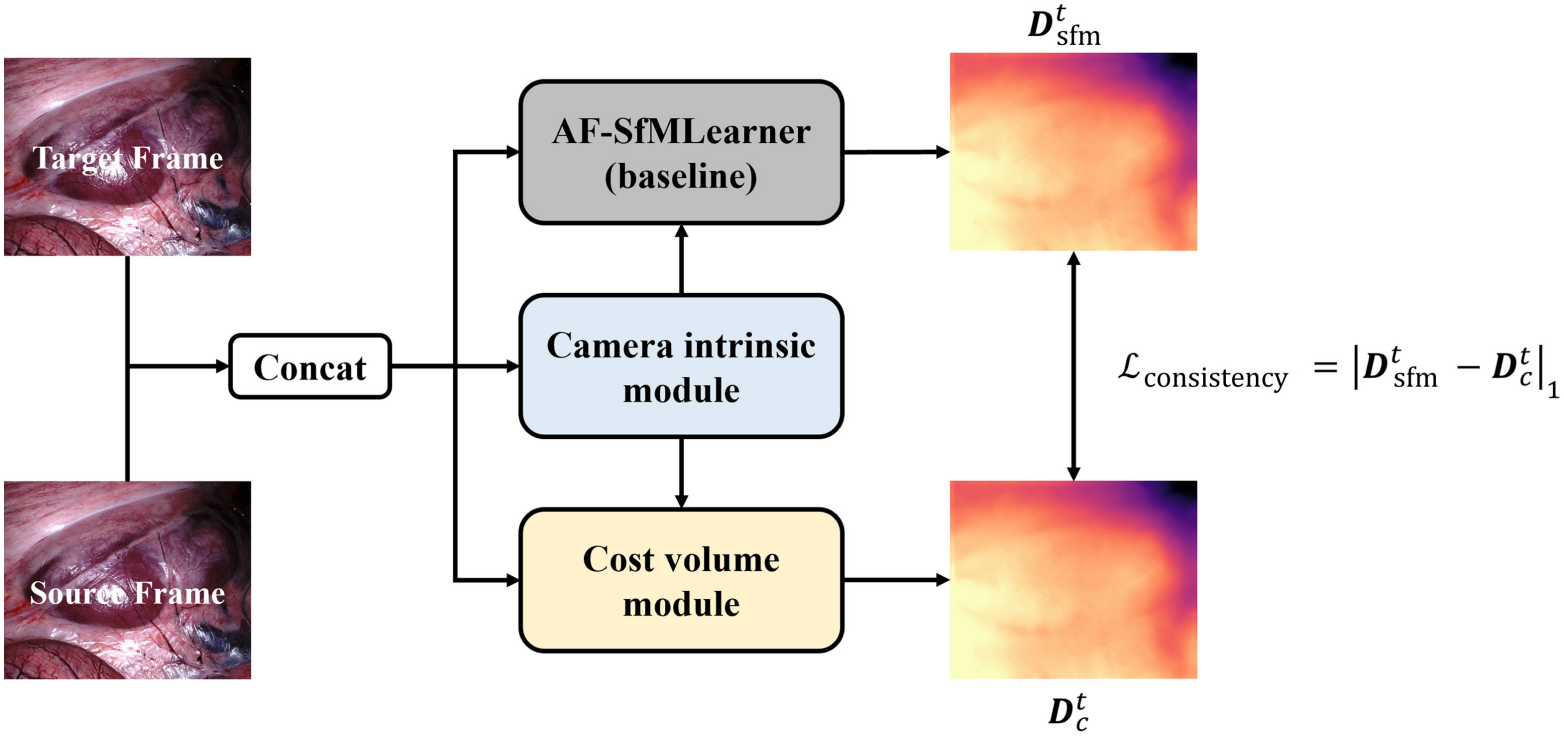
Architecture of the WS-SfMLearner.

In Secs. 3.1–3.3, we introduce the architecture of each component. In Sec. 3.4, we will outline the overall structure of our WS-SfMLearner system as shown in Fig. 5. A pair of target and source frames are sent to the camera intrinsic module to obtain the predicted camera intrinsic matrix. Then, these image pairs are fed into baseline and cost volume modules to generate depth maps $D_{\mathrm{sfm}}^{t}$ and $D_{c}^{t}$, respectively. The whole system is supervised by the consistency loss between $D_{\mathrm{sfm}}^{t}$ and $D_{c}^{t}$.

## 4 Experiments

### 4.1 Implementation Details

The proposed model is developed in PyTorch.^26^ All encoders in AF-SfMLearner, camera intrinsic module, and cost volume module use ResNet-18 pretrained on ImageNet,^27^ and the architecture of DepthNet and PoseNet is the same as the classic SfMLearner. For baseline—AF-SfMLearner—we choose α κ, $\lambda_{1}$, $\lambda_{2}$, and $\lambda_{3}$ equal to 0.85, 1, 0.01, 0.001, and 0.0001, respectively. For the cost volume module, we set $d_{\min}=0.1$ and $d_{\max}=10$. We choose the Adam optimizer with $\beta_{1}=0.9$ and $\beta_{2}=0.99$, batch size is 12, and initial learning rate is $1e^{-4}$. Learning rate is multiplied by 0.1 after every 10 epochs. The total epochs were set to 20. We set the resolution of the input image to 320×256. The whole system is training on a single RTX A5000 GPU.

The proposed model is developed in PyTorch.^26^ All encoders in AF-SfMLearner, the camera intrinsic module, and the cost volume module use ResNet-18 pretrained on ImageNet,^27^ and the architectures of DepthNet and PoseNet follow the classic SfMLearner. For all baseline hyperparameters, we follow the original settings from AF-SfMLearner. We set the loss weights to α=0.85, κ=1, $\lambda_{1}=0.01$, $\lambda_{2}=0.001$, and $\lambda_{3}=0.0001$. We use the Adam optimizer with $\beta_{1}=0.9$ and $\beta_{2}=0.99$, a batch size of 12 to ensure efficient GPU utilization, and an initial learning rate of 1e−4. The learning rate is reduced by a factor of 0.1 every 10 epochs, with a total of 20 training epochs. For the cost volume module, we set the $d_{\min}=0.1$ and $d_{\max}=10$, following the configuration from ManyDepth. The input image resolution is 320×256. Training is performed on a single RTX A5000 GPU and takes ∼8  h.

We test our novel method and compared it with other state of the arts such as SfMLeanrer,^8^ Monodepth2,^9^ HR-Depth,^28^ Li et al.,^29^ Li et al.^30^ (MICCAI’22), and AF-SfMLearner^21^ on SCARED dataset.^31^ The dataset contains nine various scenes, and we divided it at an 8:1:1 ratio for training, validation, and testing based on the video sequence.

### 4.2 Measurement Metrics

In the depth evaluation phase, we use the DepthNet from AF-SfMLearner to predict the final depth maps. Then, we scaled the depth maps with median scaling from SfMLearner^8^ which can be expressed as

$$D_{\text{scaled}}=(D_{\mathrm{pred}}*(\frac{\text{median}(D_{\mathrm{gt}})}{\text{median}(D_{\mathrm{pred}}})).$$ (8)

The scaled depth maps are capped at 200 mm on the SCARED dataset. The measurement metrics we used to evaluate the performance are listed as follows: $\mathrm{Abs}\;\mathrm{Rel}=\frac{1}{\left|D\right|}\underset{d\in D}{\sum }\frac{\left|d^{*}-d\right|}{d^{*}}$, $\mathrm{Sq}\;\mathrm{Rel}=\frac{1}{\left|D\right|}\underset{d\in D}{\sum }\frac{|d^{*}-d{|}^{2}}{d^{*}}$, $\mathrm{RMSE}=\sqrt{\frac{1}{\left|D\right|}\sum_{d\in D}|d^{*}-d{|}^{2}}$, $\mathrm{RMSE}\;\log=\sqrt{\frac{1}{\left|D\right|}\underset{d\in D}{\sum }|\log\;d^{*}-\log\;d{|}^{2}}$, and $\delta =\frac{1}{\left|D\right|}|\{d\in D|\max(\frac{d^{*}}{d},\frac{d}{d^{*}}<1.25)\}\times 100\%$ (d and $d^{*}$ denotes the predicted depth value and the corresponding ground truth, and D represents the predicted depth values).

### 4.3 Results

**Fig. 6:**
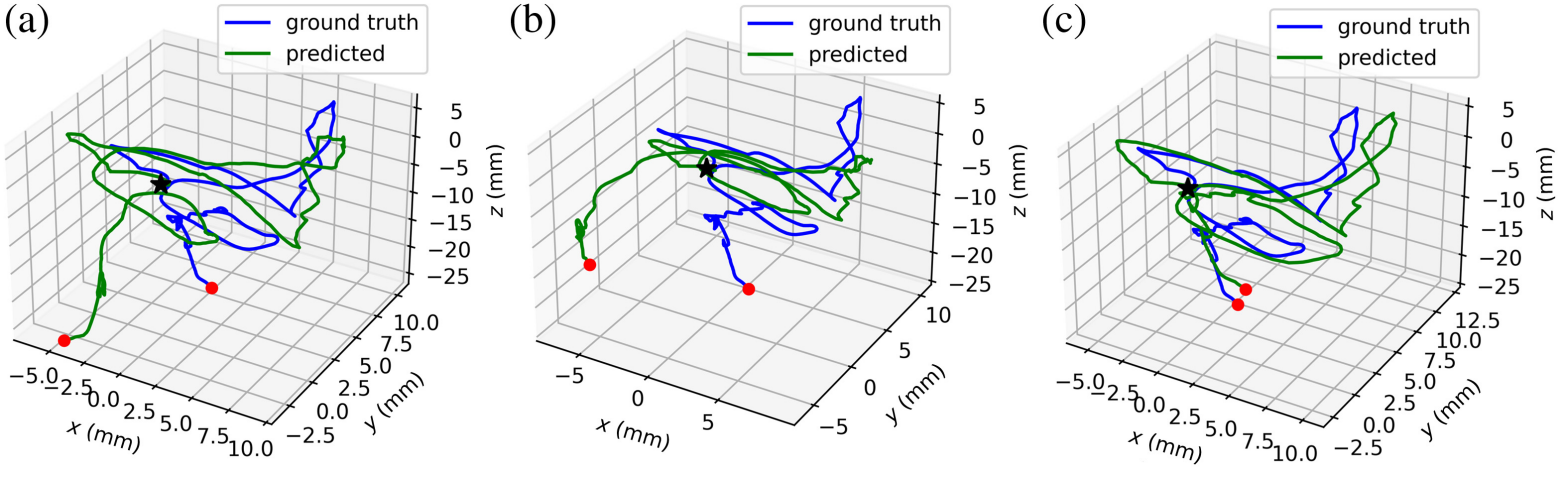
Visualization of trajectory. Trajectories predicted by (a) baseline with given intrinsic, (b) baseline with predicted intrinsic, and (c) our final version WS-SfMLearner.

Table 1 shows the quantitative results for depth estimation. The depth estimation results show even with unknown camera intrinsic parameters, our WS-SfMLearner could predict high-quality depth maps competitive with other state-of-the-art methods. In Table 2, we show the ablation studies for camera intrinsic and cost volume modules and report the ego-motion, camera intrinsic parameters, and depth estimation results. It is easy to observe that if we directly insert the intrinsic prediction module into the baseline without the cost volume, the performance declines on both depth and ego-motion estimation. After we use the cost volume module to provide auxiliary supervision, the rotation and trajectory errors decrease to less than baseline with given intrinsic parameters. In addition, we also visualize the prediction trajectory in Fig. 6, and it is clear that our proposed method helps the system predict more accurate camera motion trajectories.

**Table 1:** Comparison of the proposed model with existing models for depth estimation evaluation. “↑” and “↓” denotes higher and lower is better, respectively.

Method	AbsRel ↓	SqRel ↓	RMSE ↓	RMSElog ↓	δ<1.25 ↑	$\delta <1.25^{2}$ ↑	$\delta <1.25^{3}$ ↑
SfMLearner	0.100	2.539	11.916	0.138	0.910	0.97	7 0.993
Monodepth2	0.075	0.827	7.538	0.100	0.943	0.995	0.999
HR-Depth	0.076	0.864	7.718	0.103	0.941	0.995	1.000
Li et al.^29^	0.066	0.715	6.684	0.089	0.952	0.995	1.000
Li et al.^30^	0.062	0.654	6.649	**0.086**	0.956	**0.997**	**1.000**
AF-SfMLearner	0.065	0.575	5.763	0.093	0.957	0.995	0.999
**Ours**	**0.062**	**0.565**	**5.754**	0.089	**0.968**	0.995	0.999

Note: bold values indicate the best performance.

**Table 2:** Ablation studies for the proposed module for ego-motion, camera intrinsic parameters, and depth evaluation. For ego-motion and camera intrinsic parameter evaluation, we calculate the $l_{2}$ norm between prediction and ground truth, and results are shown in the mean ± std form. For depth estimation, we report scores of five measurement metrics (note: camera intrinsic ground truth is $f_{x}=0.82$, $f_{y}=1.02$, and $c_{x}=c_{y}=0.5$).

	Method	Baseline	Baseline + camera	Baseline + camera + cost volume
Ego motion	Rotation error	0.0433±0.0232	0.0490±0.0234	0.0400±0.0221
	Trajectory error	0.0871±0.0568	0.0996±0.0608	0.0737±0.0517
Intrinsic	$f_{x}$	—	0.86±0.005	0.81±0.003
	$f_{y}$	—	1.07±0.007	1.01±0.004
	$c_{x}$	—	0.503±0.005	0.501±0.003
	$c_{y}$	—	0.505±0.004	0.498±0.003
Depth	Abs Rel ↓	0.065	0.068	**0.062**
	Sq Rel ↓	0.575	0.616	**0.565**
	RMSE ↓	5.763	5.898	**5.754**
	RMSE log ↓	0.093	0.095	**0.089**
	δ<1.25 ↑	0.957	0.952	**0.968**

Note: bold values indicate the best performance.

## 5 Conclusion

We introduce the camera intrinsic prediction module in surgical scene depth estimation tasks to solve the problem of learning from unknown surgical videos. Next, we proposed a cost volume supervision approach. From the experimental results of depth, ego-motion, and intrinsic prediction, the proposed cost volume supervision manner improves the robustness and accuracy of the SfMLearner-based system when learning from unknown surgical videos. However, a limitation of our work is that we only evaluate the method on one large-scale dataset (SCARED) due to the lack of publicly available surgical datasets with ground truth camera intrinsics. Broader validation on additional datasets will be explored in future work. The cost volume method can still be improved by learning depth plane distribution rather than using linear distribution directly such as using sinusoidal activation function,^32^ which will be considered in our future work. In addition, addressing the challenge from moving objects, such as surgical tools within the scene, numerous segmentation methods^33^^–^^38^ have been designed to segment and recognize medical objects. Incorporating these segmentation techniques into WS-SfMLearner to alleviate dynamic uncertainties and refine depth prediction precision for the surgical background would be an interesting direction.

## Data Availability

Both code and data could be made available upon written request to the authors.
